# A Web-Based Psychoeducational Program for Informal Caregivers of Patients With Alzheimer’s Disease: A Pilot Randomized Controlled Trial

**DOI:** 10.2196/jmir.3717

**Published:** 2015-05-12

**Authors:** Victoria Cristancho-Lacroix, Jérémy Wrobel, Inge Cantegreil-Kallen, Timothée Dub, Alexandra Rouquette, Anne-Sophie Rigaud

**Affiliations:** ^1^Assistance Publique - Hôpitaux de ParisDepartment of GerontologyHôpital BrocaParisFrance; ^2^EA 4468. Maladie d'Alzheimer, facteurs de risques, soins et accompagnement des patients et des famillesUniversité Paris DescartesParisFrance; ^3^Assistance Publique - Hôpitaux de ParisDepartment of Biostatistics and EpidemiologyHôpital Hôtel-DieuParisFrance; ^4^Institut national de la santé et de la recherche médicaleUnité Mixte de Recherche-S0669Université Paris-Sud and Université Paris DescartesParisFrance; ^5^Research Unit on Children’s Psychosocial MaladjustmentUniversity of MontrealMontréal, QCCanada

**Keywords:** family caregivers, psychological education, eHealth, Alzheimer disease, emotional stress, qualitative research, Internet, randomized controlled trials

## Abstract

**Background:**

Although several face-to-face programs are dedicated to informal caregivers of persons with dementia, they are not always accessible to overburdened or isolated caregivers. Based on a face-to-face intervention program, we adapted and designed a Web-based fully automated psychoeducational program (called Diapason) inspired by a cognitive approach.

**Objective:**

This study aimed to evaluate through a pilot unblinded randomized controlled trial the efficacy and acceptability of a Web-based psychoeducational program for informal caregivers of persons with Alzheimer’s disease (PWAD) based on a mixed methods research design.

**Methods:**

We recruited and randomized offline 49 informal caregivers of a PWAD in a day care center in Paris, France. They either received the Web-based intervention and usual care for 3 months (experimental group, n=25) or only usual care (control group, n=24). Caregivers’ perceived stress (PSS-14, primary outcome), self-efficacy, burden, perceived health status, and depression (secondary outcomes) were measured during 3 face-to-face on-site visits: at baseline, at the end of the program (month 3), and after follow-up (month 6). Additionally, semistructured interviews were conducted with experimental group caregivers at month 6 and examined with thematic analysis.

**Results:**

Intention-to-treat analysis did not show significant differences in self-perceived stress between the experimental and control groups (*P*=.98). The experimental group significantly improved their knowledge of the illness (*d*=.79, *P*=.008) from baseline to month 3. Of the 25 participants allocated to the experimental group, 17 (71%) finished the protocol and entirely viewed at least 10 of 12 online sessions. On average, participants used the website 19.72 times (SD 12.88) and were connected for 262.20 minutes (SD 270.74). The results of the satisfaction questionnaire showed that most participants considered the program to be useful (95%, 19/20), clear (100%, 20/20), and comprehensive (85%, 17/20). Significant correlations were found between relationship and caregivers’ program opinion (*P*=.01). Thus, positive opinions were provided by husbands and sons (3/3), whereas qualified opinions were primarily reported by daughters (8/11). Female spouses expressed negative (2/3) or neutral opinions (1/3). Caregivers expected more dynamic content and further interaction with staff and peers.

**Conclusions:**

In this study, quantitative results were inconclusive owing to small sample size. Qualitative results indicated/showed little acceptance of the program and high expectations from caregivers. Caregivers did not rule out their interest in this kind of intervention provided that it met their needs. More dynamic, personalized, and social interventions are desirable. Our recruitment issues pointed out the necessity of in-depth studies about caregivers’ help-seeking behaviors and readiness factors.

**Trial Registration:**

Clinicaltrials.gov NCT01430286; http://clinicaltrials.gov/ct2/show/NCT01430286 (Archived by WebCite at http://www.webcitation/6KxHaRspL).

## Introduction

Due to the worldwide aging population, the number of persons with dementia (35.6 million currently) is expected to double by 2030. The socioeconomic consequences of this rapid rise and the absence of an effective pharmacotherapy have positioned dementia as a major public health concern in recent years [1]. The Alzheimer’s Association reported that in 2012 more than 15 million caregivers provided an estimated 17.5 billion hours of unpaid care, representing US $216 billion [2]. Today, the majority of persons with Alzheimer’s disease (PWAD) living at home are cared for by their spouses, children, or friends [3]. Nevertheless, the amount of time dedicated to their relative, the physical efforts, and the strong emotional involvement associated with caregiving may induce chronic stress in caregivers and weaken their physical and mental health [4-7]. Such repercussions can also negatively affect other areas of their lives (eg, professional or social) [8].

Various nonpharmacological intervention programs for caregivers are available on-site (ie, [9,10]). Nevertheless, some caregivers are not willing or available to attend a face-to-face program due to a lack of respite, the distance, or owing to care-recipients’ behavioral or physical problems. For them, technology-based programs may represent an interesting complementary strategy to regular care management [11,12].

Based on a face-to-face psychoeducational program [13], we adapted and developed the Diapason program, based on a user-centered design, including a proof of concept and 2 usability tests [14]. Although other recent Internet-based programs have been tested [15,16], to our knowledge, the use of mixed research methods still remains rare in randomized controlled trials (RCTs) [17]. Yet, including qualitative analysis in the evaluation of these programs may improve results interpretation, help “trialists” become more sensitive to individual differences, and save money “by steering researchers toward interventions more likely to be effective in future trials” [18].

The main aim of this pilot RCT was to evaluate the impact of the Diapason program on caregivers’ perceived stress. We hypothesized that this program offering information, skills training, and a forum for caregivers would significantly reduce their perceived stress and burden, and enhance caregivers’ self-efficacy, self-perceived health, and self-perceived knowledge about the disease. Qualitative analyses would facilitate the identification of subgroups benefiting from the program and would guide us to improve content and methods to evaluate this type of intervention.

## Methods

### Study Design

We carried out an unblinded monocentric pilot RCT (NCT01430286) between 2011 and 2014 in a day care center geriatric unit (Paris, France). Informed consent was obtained before participation. French ethical CPP approved this protocol in July 2011. The in-depth description of the protocol study has been reported elsewhere [19].

### Recruitment and Participants

The recruitment strategy included flyers and posters placed in the hospital. During the consultations, geriatricians proposed this protocol to caregivers of PWAD. The caregivers interested in the study filled out a contact form. Then a psychologist provided them with the information form, confirmed inclusion criteria, and collected the signed informed consent.

Eligible participants were required to be French-speaking caregivers of community-dwelling PWAD who met the criteria of the *Diagnostic and Statistical Manual of Mental Disorders*, 4th Edition [20]. Caregivers had to spend at least 4 hours per week with their relative, be aged 18 years or older, scored 12 or more on the Perceived Stress Scale (PSS-14), and to have access to a computer with Internet connection. Professional caregivers were ineligible.

Based on the literature, a 6-point difference on PSS-14 was expected between the experimental and control groups at 3 months [21]. With an assumed SD of 9, 40 participants per group needed to be included to detect this difference with an 80% power (Cronbach alpha=.05; 2-tailed).

### Intervention

**Figure 1:**
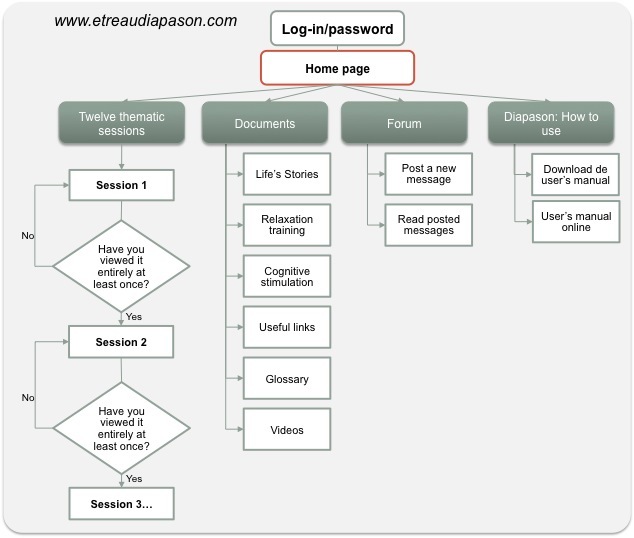
Layout of the Diapason program and process for viewing weekly sessions.

The Diapason program [22] was delivered in a free, password-protected, fully automated website to be used in an individual fashion, at home, by the caregivers. The program’s content was based on cognitive theories of stress, a literature review [23], and the results of a study conducted by our team [13]. In the latter, caregivers who improved their understanding of cognitive and behavioral symptoms reported feeling less stressed. Furthermore, caregivers with a perceived personal time restriction or poor social support suffer more stress, burden, and depression [24,25]. Consequently, our intervention targeted (1) caregivers’ beliefs about the illness and the caregiving role, (2) caregivers’ skills to manage daily life difficulties, and (3) caregivers’ social support and help-seeking behavior to obtain respite or financial support, and to meet and discuss with peers through a forum. Twelve thematic sessions were sequentially and weekly unblocked once the previous one was entirely viewed (see layout in Figure 1). Owing to the variability of behavioral and psychological symptoms depending on the type of dementia and the important impact of some of them on caregivers’ stress (eg, hallucinations, delusions), only Alzheimer’s disease was targeted by this program.

Each session included theoretical and practical information, videos of health professionals, and a practice guide for applying the session’s content in real life. The length of the intervention was 3 months, with each weekly session lasting 15 to 30 minutes on average, but there was no time limit and the participants could access different website sections (eg, relaxation training, forum) for as long as they wished at any time. The program content is summarized in Textbox 1.

**Overview of Diapason program contents.**

Weekly Sessions

One session per week had to be entirely viewed at least once to unblock the next session

Session 1. Caregiver stress: this session presents a definition of stress, its causes and consequences on caregivers, the risk factors for chronic stress, and the mechanisms and effects of relaxation (includes a link to the relaxation training in the Diapason website), as well as strategies for managing stress underlining the importance of looking for respite.

Session 2. Understanding the disease: in this session, the Alzheimer’s disease diagnosis procedure, the symptoms, the progression of the illness, and the consequences on daily life activities for persons with Alzheimer’s disease (PWAD) are explained.

Session 3. Maintaining the loved ones’ autonomy: this session presents the reasons and strategies to involve loved ones in the process of care in order to stimulate the preserved functions and compensate for the lost ones. The session underlines the importance of maintaining the self-esteem of PWAD.

Session 4. Understanding their reactions: in this session, the most frequent behavioral and psychological symptoms of dementia (BPSD) and their characteristics are succinctly described and illustrated by examples from daily life. The contextual and intrinsic factors that might be associated with them are also described.

Session 5. Coping with behavioral and emotional troubles: this session presents practical advice on how to cope vis-à-vis the BPSD described in the previous session.

Session 6. Communicating with loved ones: this session includes the description of the most frequent language troubles and the strategies to modulate and adapt communication to the preserved skills of PWAD.

Session 7. Improving their daily lives: this session presents strategies to facilitate the performance of activities that become difficult or impossible to execute due to apraxia, illustrating them with examples adapted to daily life.

Session 8. Avoiding falls: the session includes practical advice for maintaining and stimulating the relative’s balance and actions to adopt in the event of a fall. In addition, various actions are described to adapt the relative’s home.

Session 9. Pharmacological and nonpharmacological interventions: this session includes a brief presentation of different interventions available for caregivers in France with pharmacological treatment as well as cognitive and psychological support.

Session 10. Social and financial support: this session presents the different stakeholders and services that may help caregivers in their daily life. The financial and social support provided by the French government is also overviewed.

Session 11. About the future: this session provides caregivers with information about the role of disease progression anticipation, inviting them to try and foresee solutions keeping a prospective vision, encouraging them to look for further sources of information, and social support to reduce the uncertainty of caregiving situations.

Session 12. In a nutshell: the last session encompasses a summary of the Diapason program, emphasizing the acceptance of support and help and the importance of obtaining more information to anticipate and avoid stressful circumstances.

Additionally the website contains other sections that can be consulted at any time.

Relaxation training: guidelines for learning relaxation as well as 2 videos for the modeling of Schultz’s Autogenic Training and Jacobson’s method.

Life Stories: stories about 4 couples, based on testimonials of caregivers, in which difficult situations are illustrated and possible solutions to manage them are discussed (eg, apathy of patient, caregivers’ isolation).

Glossary: a glossary for technical words (eg, neuropsychological assessment, aphasia)

Stimulation: practical activities to stimulate autonomy and share pleasant activities with the relatives in daily life.

Forum: a private and anonymous forum to interact with peers, to express their concerns, discuss solutions to daily problems, and share their feelings and experiences. The participants use nicknames to protect their privacy. A clinical psychologist participates in the discussions if necessary (ie, avoiding aggressive or inappropriate comments).

### Procedure

Participants were recruited and randomized offline in 2 parallel groups based on a computer-generated randomization list using blocking and stratification by sex and relationship (spouses vs nonspouses).

The experimental group participants received at baseline a 10-minute training session on how to use the website, a log-in and password, a printed version of the user’s manual, and a notebook to write personal ideas about their application of the program’s content. Each week, participants had to read through an entire thematic session and fill out a printed satisfaction questionnaire. Other website sections (eg, relaxation training, forum) were available but not mandatory to complete the program. No modification regarding methodology, program content (except for forum discussions), or the website was done during the course of the study.

The control and experimental group participants received usual care, in which they were provided with information about the illness during their semiannual follow-up with their geriatrician. The control group participants were given access to the Diapason program at the end of their participation. All participants were advised to look for additional help if necessary and were asked to inform the researcher about it.

An individual face-to-face assessment was conducted at the Broca hospital by research psychologists (VCL or JW) at baseline, at the end of the program (at month 3), and after 3 months of follow-up (at month 6). Each 90-minute assessment visit consisted of a structured interview, standardized questionnaires, and visual analog scales (VAS). Additionally, experimental group volunteers participated in an optional one-to-one semistructured interview at the 3-month follow-up.

### Measures

Based on a cognitive approach inspired by Lazarus and Folkman’s [26] stress and coping theory and Bandura’s [27] self-efficacy model, we hypothesized the program would have a direct impact on perceived stress levels, self-efficacy, and burden, and may influence depression and self-perceived health status.

To evaluate the perceived stress of caregivers (primary outcome), we used the 14-item Perceived Stress Scale (PSS-14) [28]. The total score ranges from 0 to 56, higher scores representing higher stress levels. In order to target the caregiving stress, we adapted the instruction by proceeding with heteroevaluation and adding the following underlined text: “This scale asks you about your feelings and thoughts about your experience with your relative during the last 4 weeks.”

The secondary outcomes were:

1. Self-efficacy measured by the Revised Scale for Caregiving Self-Efficacy (RSCS) [29], which distinguishes 3 self-efficacy domains: obtaining respite, responding to disruptive behavior, and controlling upsetting thoughts. Scores in each domain range from 0 to 100, higher scores indicating a higher degree of confidence for each situation.
2. Perception and reaction to cognitive or behavioral symptoms of PWAD were evaluated with the Revised Memory and Behavior Problems Checklist (RMBPC) [30]. This instrument rates 24 problems on 2 scales. They evaluate (5-point scale) frequency and caregiver’s bother or strain for each problem. A global score ranging from 0 to 4 was calculated for both scales. Higher scores indicate higher frequency or higher emotional effects.
3. Subjective burden was evaluated with the French version of the Zarit Burden Interview [31]. The total score ranges from 0 to 88, a higher score meaning a higher burden level.
4. Depressive symptoms were measured with the second version of the Beck Depression Inventory (BDI-II) [32] including 21 items with a total score range from 0 to 63. Higher scores indicate higher levels of depressive symptoms.
5. Self-perceived health was measured with the French version of the Nottingham Health Profile (NHP) [33]. We analyzed social isolation, emotional reactions, and sleep quality subscores and rated each from 0 to 100, which provided a percentage of the perceived illness impact.

At each visit, we collected information on caregiving variables (structured questionnaire). On the 4 VAS, caregivers evaluated their current levels (from 0=low to 100=high) of (1) knowledge about Alzheimer’s disease, (2) overall stress, (3) self-efficacy for coping with the illness, and (4) the caregivers-PWAD relationship quality.

Web metrics (session length and rate of visits) were collected for each experimental group participant automatically and anonymously. Participants completed a weekly satisfaction questionnaire focused on utility, clarity, and comprehensiveness (5-point Likert scale). They rated from 0 to 100 the applicability and positive emotional impact of each session and reported their opinion of the program (open-ended question). At the end of their participation, we proposed a semistructured interview exploring their opinion of the program.

Concerning the PWAD, we collected at baseline the Mini-Mental State Examination (MMSE) [34] from the medical record and Instrumental Activities of Daily Living (IADL [35]) and the date of symptom onset (reported by the caregiver).

### Data Analysis

All available data at baseline were analyzed by intention-to-treat analysis. Descriptive statistics (means and percentages) were calculated for caregivers’ and PWAD’s characteristics. Moreover, *t* tests (or Mann-Whitney tests) and Spearman or polyserial correlations were used to assess associations between variables. The missing data within each scale were treated according to the recommendations of the literature when available. Otherwise, simple mean imputation was used. The last observation carried forward method was used for participants who dropped out. After checking normality and homoscedasticity of primary outcome (PSS-14), we conducted an analysis of covariance (ANCOVA), controlling for regression to mean phenomenon and effects of potential confounders at baseline on primary outcome. All analyses were conducted using *R* Software for Windows (version 3.0.0).

Interviews and open-ended questions were concurrently analyzed by two trained psychologists (JW and VCL) following the thematic analysis method, using a semantic approach, driven by analytic interests and an essentialist/realist approach [36].

## Results

### Participants

**Figure 2:**
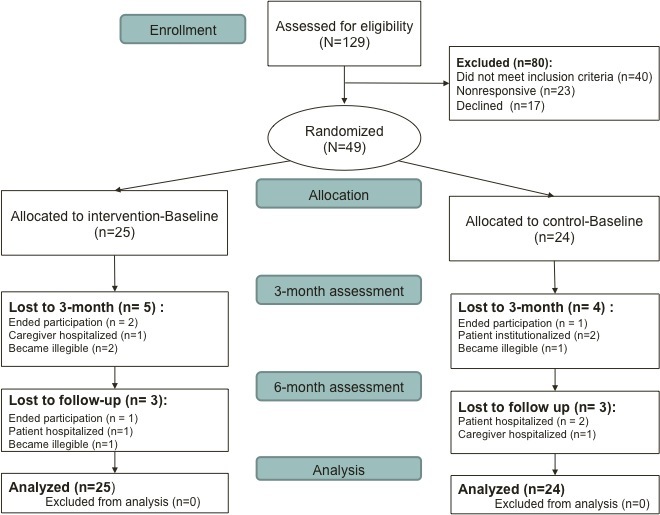
Flowchart of the Diapason pilot randomized controlled trial.

As summarized in the flowchart (Figure 2), of the caregivers met by the physicians, 129 were prescreened between December 2011 and August 2013. Among them, 40 did not meet inclusion criteria (ie, did not use the Internet, did not accept/know the diagnosis, were not available for 3 assessments at the hospital), 23 were unreachable, and 17 declined. After an 8-month recruitment extension, the main investigators (ASR and VCL) stopped recruitment (in total 20 months) because the rate of inclusions did not exceed 2 persons per month on average.

We randomized 49 participants. Of the 25 participants allocated to the experimental group, 17 (71%) finished the protocol and validated at least 10 of 12 online sessions. Four participants ended their participation in the study without withdrawing consent.

Demographics and other characteristics of participants are summarized in Table 1. At baseline, the groups were imbalanced regarding the number of weekly hours of professional help and IADL and BDI-II scores. The PSS-14 scores were correlated with weekly professional help received (ρ=.33) and BDI-II scores (ρ=.49), whereas the correlation with IADL scores was weak (ρ=–.11).

**Table 1:** Demographics and key characteristics at baseline by group (N=49).

Characteristics		Experimental group	Control group
**Caregivers’ characteristics, n**		25	24
	Caregiver age (years), mean (SD)	64.2 (10.3)	59.0 (12.4)
	Female caregiver, n (%)	16 (64)	16 (67)
	Children of PWAD,^b^ n (%)	16 (64)	13 (54)
	High level of education, n (%)	19 (76)	18 (75)
	Middle level of education, n (%)	6 (24)	3 (12)
	Living with the PWAD, n (%)	12 (48)	10 (41)
	Visiting the PWAD daily, n (%)	4 (16)	2 (8)
	Visiting the PWAD at least once per week, n (%)	9 (36)	9 (38)
	Psychological/ psychiatric treatment, n (%)	3 (12)	2 (8)
	Psychotropic treatment, n (%)	6 (24)	7 (29)
	Caregivers with at least another source of stress different to caregiving (eg, work, relationship, family), n (%)	18 (72)	14 (56)
	Caregivers with ≥1 professional help,^c^ n (%)	18 (72)	18 (75)
	Weekly hours of professional help,^d^ mean (SD)	26.7 (28.7)	8.2 (9.7)
	Suffering from a chronic pathology, n (%)	9 (36)	8 (33)
**Patients’ characteristics, n**		25	24
	Onset of symptoms (years), mean (SD), range	4.62 (3.53), 0.55-14.05	4.11 (3), 0.39-12.03
	MMSE, mean (SD)	18.5 (5.4)	19.0 (4.6)
	IADL scale, mean (SD)	0.6 (0.8)	1.1 (1.1)

^a^ IADL: Instrumental Activities of Daily Living; MMSE: Mini-Mental State Examination; PWAD: persons with Alzheimer’s disease.

^b^ Two participants were not children or spouses (1 daughter-in-law and 1 friend).

^c^ Professional help=housekeeper, nurse, day care, meal delivery.

^d^ Among caregivers receiving respite help.

### Primary Outcome: Self-Perceived Stress

Mann-Whitney tests did not show significant differences between the experimental and control groups over time (Table 2). We conducted ANCOVA with the PSS-14 at month 3 as dependent variable and the PSS-14 at baseline, group, stratification factors (sex and relationship), and potential confounders at baseline (BDI-II and professional help received) as independent variables. Only the PSS-14 at baseline (*P*<.001) and weekly help received (*P*=.01) were significantly associated with PSS-14 at month 3. Thus, no significant relationship was found with the intervention (*P*=.34). ANCOVA showed similar results when stratification factors were not included.

**Table 2:** Outcome measures (means and SDs) for assessments at baseline (M0), after intervention (M3), and at 6-month follow-up (M6) per group.

Scales and subscores^a^		Experimental, mean (SD)				Control, mean (SD)				*P* ^b^
		M0	M3	M6	Diff^c^	M0	M3	M6	Diff^c^	
PSS-14		24.2 (9.0)	23.7 (9.2)	25.0 (9.9)	–0.5 (8.0)	24.5 (6.7)	23.8 (6.2)	23.8 (6.9)	–0.7 (4.5)	.98
**RSCS**										
	Obtaining respite	55.0 (26.9)	51.7 (29.3)	54.7 (30.6)	–3.3 (18.3)	49.2 (22.4)	48.9 (26.8)	48.6 (24.5)	–0.4 (24.1)	>.99
	Responding to patients’ behaviors	72.2 (17.0)	69.0 (19.7)	71.5 (23.1)	–3.2 (14.1)	66.3 (18.2)	65.8 (22.7)	68.4 (15.3)	–0.5 (15.5)	.52
	Controlling upsetting thoughts	62.6 (21.3)	63.2 (19.7)	63.4 (20.8)	0.5 (17.0)	64.7 (18.1)	66.3 (14.9)	64.0 (13.7)	1.5 (16.1)	.83
**RMBPC**										
	Frequency	1.6 (0.5)	1.8 (0.6)	1.8 (0.6)	0.1 (0.4)	1.5 (0.6)	1.6 (0.6)	1.6 (0.7)	0.0 (0.3)	.72
	Reaction	2.2 (0.4)	2.2 (0.6)	2.3 (0.5)	0.0 (0.4)	2.2 (0.6)	2.1 (0.6)	2.1 (0.6)	–0.1 (0.5)	.66
ZBI		38.0 (14.5)	38.3 (14.9)	39.6 (15.7)	0.3 (6.6)	35.0 (15.0)	33.5 (15.3)	34.8 (15.9)	–1.5 (6.1)	.74
BDI-II		11.2 (10.1)	11.5 (9.2)	12.4 (11.6)	0.3 (4.6)	9.0 (7.4)	8.9 (6.5)	8.8 (7.2)	–0.1 (2.7)	.56
**NHP**										
	Social isolation	14.1 (20.4)	15.9 (21.7)	16.5 (23.4)	1.9 (9.7)	12.5 (17.2)	15.5 (19.9)	14.8 (20.7)	3.0 (14.9)	.79
	Emotions	20.6 (22.4)	18.6 (18.09)	26.6 (25.6)	–2.1 (16.4)	18.6 (20.3)	19.0 (19.5)	17.2 (19.2)	0.4 (12.9)	.84
	Energy	27.9 (39.1)	25.3 (33.6)	35.9 (39.4)	–2.6 (30.6)	26.6 (31.7)	38.5 (38.8)	35.6 (41.6)	11.9 (34.2)	.22
**VAS**										
	Knowledge	45.4 (23.2)	59.2 (25.9)	58.6 (24.4)	13.8 (15.1)	44.5 (23.5)	44.4 (21.6)	51.7 (18.8)	–0.0 (17.4)	.008
	Coping	67.4 (15.8)	67.6 (13.3)	67.2 (17.6)	–0.2 (13.8)	61.4 (21.8)	61.4 (15.7)	61.8 (17.5)	0.0 (16.5)	.71
	Stress	40.7 (23.0)	48.6 (24.3)	50.6 (23.2)	7.9 (23.8)	50.2 (15.3)	46.7 (16.7)	50.3 (17.0)	–3.5 (16.5)	.05
	QR	71.4 (20.5)	73.8 (21.5)	72.7 (17.9)	2.4 (13.5)	72.1 (16.9)	69.0 (23.8)	69.3 (18.0)	–3.0 (19.5)	.36

^a^ BDI-II: Beck Depression Inventory-second version; NHP: Nottingham Health Profile; PSS-14: self-perceived stress; QR: quality of relationship between caregiver and the patient; RMBPC: Revised Memory and Behavior Problem Checklist; RSCS: Revised Scale for Caregiving Self-Efficacy; VAS: visual analog scale; ZBI: Zarit Burden Interview.

^b^ Comparing means differences (M3 – M0) of experimental and control groups by Mann-Whitney tests.

^c^ Means difference (M3 – M0).

### Secondary Outcomes

Only the VAS evaluating knowledge of the disease showed significant change at month 3 scored a high effect size (Cohen’s *d*=.79, *P*=.008). Indeed, the experimental group scores increased by 13.8 points (SD 15.1), whereas the control group scores decreased by 0.04 points (SD 17.4) (Table 2). However, no significant differences were found between the groups from baseline to month 6.

Only one user reported problems watching the videos (Flashplayer was not installed on computer) and another with little experience using the Internet could not use it unaided. The high scores on the weekly satisfaction questionnaire showed that nearly all participants considered Diapason topics to be useful (95%, 19/20), clear (100%, 20/20), and comprehensive (85%, 17/20). Topics describing strategies to maintain relatives’ autonomy and coping skills with the PWAD’s behavioral troubles fostered higher levels of positive emotional impact (mean 61.50, SD 22.83 and mean 61.90, SD 26.68, respectively). The most applicable session was focused on coping skills of the PWAD’s behavioral troubles (mean 72.25, SD 15.22). In contrast, the session describing caregiving stress factors and protectors received the lowest scores for positive emotional impact (mean 49.25, SD 21.75) and applicability (mean 61.00, SD 17.67).

On average, participants used the website 19.72 times (SD 12.88) and for 262.20 minutes (SD 270.74) during the first 3 months. The most frequently visited section was the forum (mean 24.86 times, SD 40.95), whereas only 10 messages and 10 answers were posted. Four spouses (45%) and 4 daughters (33%) visited the website 26 times or more (third quartile). No significant correlation was found between the PSS-14 score (M3–M0) and frequency (ρ=–.15) or duration (ρ=–.05) using the website. After month 3, connection times were near zero.

### Qualitative Analysis

**Figure 3:**
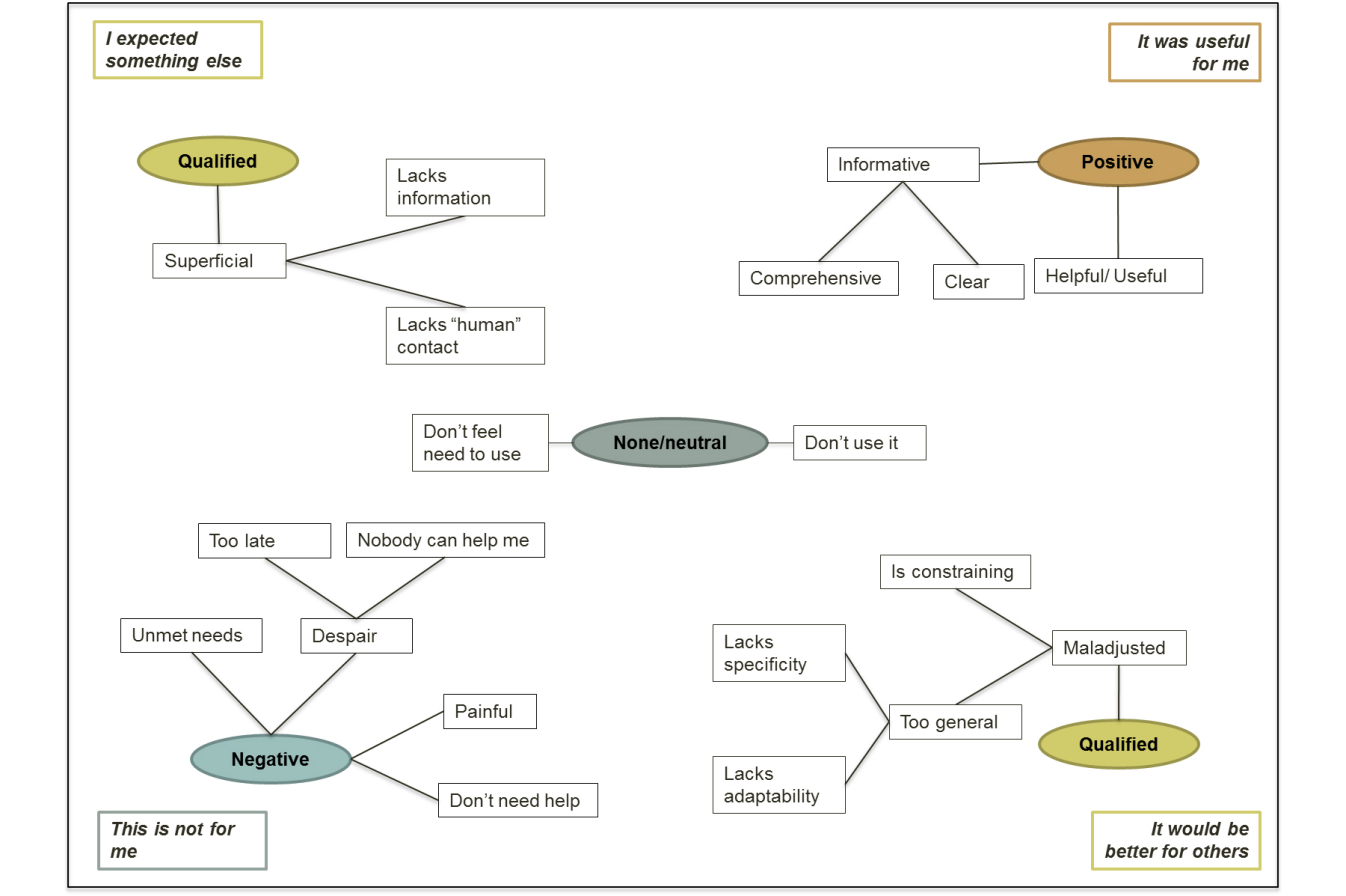
Thematic map of opinions and reasons given by users.

Thematic analysis on the participants’ impressions underlined four trends: caregivers without a clear opinion toward the program (5/25, 20%) and those with a clearly positive (3/25, 12%), qualified (11/25, 44%) or negative (6/25, 24%) opinion. These trends were significantly associated to the relationship (Fisher’s exact test, *P*=.01). Thus, most wives had a negative opinion, whereas daughters primarily expressed a qualified opinion about the program. Only male caregivers expressed a positive opinion (see Table 3). As shown in Figure 3, reasons varied between caregivers of a single category.

**Table 3:** Caregivers’ profiles and opinions about the Web-based program (N=25).

Demographics		None	Negative	Qualified	Positive
Age, mean (SD)		58.00 (4.24)	66.83 (11.81)	62.45 (9.36)	72.00 (13.45)
**Relationship, n**					
	Wife	1	3	0	0
	Husband	1	1	1	2
	Daughter	2	2	8	0
	Son	1	0	2	1
Total, n (%)		5 (20)	6 (24)	11 (44)	3 (12)

Moreover, we distinguished 4 topics comprising caregivers’ opinions (examples in Table 4):

1. “It was useful for me.” A few participants reported having benefited from the program. They said it improved their understanding on the disease or changed their initial beliefs about the disease or diagnosis*.*
2. “It would be better for others.” Participants considered the program would be better attuned to the needs of a PWAD in other (earlier or more advanced) stages of the disease than their relatives. Most children thought the overall “message” was more adapted for spouses rather than for them. The contrary was not stated.
3. “I expected something else.” Some participants found the content was not in-depth enough. They expected more specific and individualized advice, and more “human interaction” with professionals or peers.
4. “This is not for me.” Other participants preferred another kind of intervention (eg, individual therapy, respite, financial support) or reported not feeling a need for help. Others considered the program had come too late or did not believe that someone/something could help them. Most of them ended their participation.

Additionally, many experimental and control group participants reported having used other resources to better understand the disease and adapt their behavior (eg, reading books, asking for help, or contacting associations).

**Table 4:** Categories and examples of qualitative data.

Topic	Example quote (verbatim)
It was useful for me	Mr. P, husband, 83 y/o: “The topics were highly interesting and useful for me. Advice is clear and helpful for improving communication with my wife”
	Mr. R, son, 51 y/o: “The more I read the more I found it interesting. Sometimes I came back (to the first sessions) and I found that my perception of the topics had changed (...) I’ve understood that my mother behaves like this because of the illness, and her reactions are not against me”
	Mr. L, husband, 80 y/o: “At the beginning I did not feel concerned, I was wrong. Maybe I was in denial. Now I find (in the program) a lot of interesting advice”
The program would be better for others	Mrs. L., daughter, 55y/o: “I did not feel concerned at all, not yet (...) my mother is in the earliest stages”
	Mrs. R, wife, 75 y/o: “This program is not adapted to the current state of my husband, he was diagnosed 7 years ago, I’ve already experienced these situations”
	Mrs. FR, daughter, 55 y/o: “(...) some ideas and solutions are more adapted for spouses or for someone living with the person”
I expected something else	Mrs. L, daughter, 56 y/o: “The content is almost superficial, it lacks more information about books, addresses, events (...)”
	Mrs. R, daughter, 55 y/o: “I wished to know how to accurately behave or react when my mother upsets me, when she repeats the same question”
	Mr. L, husband, 81 y/o: “(Diapason) is too impersonal and “cold,” I tried to use the forum, but I need to look at the person in front of me (...)”
This is not for me	Mr. C, husband, 71 y/o: “I still don’t understand why the doctor said she had Alzheimer’s. For me she is depressed, that is all, this is normal after retirement (...)”
	Mrs. C, daughter, 56 y/o: “I know how to manage my mother, I have acquired some more experience in my professional life (Professor in Economics) The most important is to be organized, I am not stressed (...) the reason why I’ve participated is only to contribute to research”
	Mrs. M, daughter, 60 y/o: “I’ve tried to use the website, but reading how my mother will lose her memory, her abilities is painful for me, (...) I am anxious, I’d preferred a psychotherapy. Finally I am not ready for that (...)”

## Discussion

### Principal Findings

Statistical analysis did not show significant differences in self-perceived stress (PSS-14) between the groups over time. This result is most likely due to low statistical power. Perceived stress levels remained stable over time in PSS-14 scores although the disease progressed. This stabilization has been observed in control groups from similar studies, suggesting that caregiving stress rarely increases over a period of 3 months [16]. After 6 months, a few experimental group participants had heightened stress levels. This may be due to a raised awareness of their loved one’s diagnosis. Even if it is a major source of stress, being aware of diagnosis might help caregivers to deploy adapted coping strategies (eg, self-regulation, problem-focused coping, positive emotion) [37], whereas those using avoidance-escape strategies (eg, denial of diagnosis) may suffer from more negative long-term consequences (eg, inability to cope with behavioral problems) [38].

As in other studies [13], the experimental group participants’ self-perceived level of disease knowledge was significantly improved between baseline and month 3, with control group participants reaching similar levels at month 6. During the first 3 months, the program may have accelerated the learning process, but the control group may have improved their perception of disease knowledge at month 6 based on their experience and information from other sources (eg, websites, books, professionals/institutions, friends).

During the first 3 months, the program was highly used, in contrast with other studies [39], most likely due to mandatory reading of weekly sessions. Nevertheless, once the program was finished (after 3 months) almost none of the participants used the website, probably due to the stasis of the program’s content. No significant correlation was found between frequency or duration of website use and stress levels (PSS-14).

### Qualitative Results

Our qualitative findings are comparable to previous works. Caregivers considered the program could be useful for people other than themselves [11]. They wished to receive personalized support, extensive information, specific assistance, and more communication with professionals and peers [40]. They preferred the topics offering strategies to maintain the PWAD’s autonomy and teaching skills for coping with behavioral problems [41], but were less interested in self-care [7]. Furthermore, specific subgroups of caregivers benefited from the program [42,43]. Some reported having a better perception of the disease or accepted diagnosis after the program [13]. In contrast with other studies [11], the most interested users were male caregivers. Probably linked to their preference for information and skills-centered interventions (such as Diapason) rather than emotional-focused ones [44]. In our study, daughters expressed more qualified opinions about the program compared to female spouses. In our view, because children caregivers are often active workers, they may recognize distance-based interventions as an interesting alternative for them. Moreover, female spouses facing greater caregiving challenges may be less aware of caregiving consequences for themselves [45] and may need more personalized interventions.

### Strengths of the Study

To our knowledge, this is the first pilot RCT based on a mixed methods research design evaluating an online program for caregivers of PWAD. By using a mixed method research design, this program follows current methodological trends [46] using qualitative data to complement and contextualize RCT results [18]. Based on literature recommendations [47], Diapason adopted a multicomponent structure combining information and interaction between caregivers. Furthermore, this study met almost all the “best practice” criteria for a RCT (ie, randomization, intention-to-treat analysis, prior sample size calculation, and restriction of analysis to primary outcomes) [9] and controlled the intervention’s implementation errors [48]. Indeed, we paid particular attention to control implementation error. For instance, we controlled the information viewed by the caregiver according to a specific schedule. Additionally, the website content remained static during the study offering the same content to all participants. Finally, in order to avoid bias associated with the hypothetical imbalance of number of messages exchanged with the professional at the beginning and at the end of the protocol (eg, the first participants would not have benefited from discussions published later in the study), professionals only acted as moderators.

### Limits and Lessons Learned

In spite of using different strategies, the recruitment for this study was difficult; only 38.0% (49/129) of prescreened caregivers were actually enrolled. These difficulties occur in Internet-based intervention studies [16], suggesting it may be due to caregivers’ attitudes toward these programs [49]. Nevertheless, their reluctance to face-to-face services was also described [49-51]. Thus, further studies about caregivers’ help-seeking behaviors and readiness facilitators or predictors are warranted [52].

Although face-to-face trials allowed the control of bias, isolated caregivers and those living in remote regions could participate more easily if the trials were conducted online only. In any case, replication with larger samples is necessary to complement our results. In addition, due to the heterogeneity of caregiver populations, we advise limiting the number of inclusion criteria [42] and the number of variables measured to reduce analysis bias. Finally, we pointed out the risk of bias owing to nonblinded assessments in this study [53].

This pilot study evaluated the first online version of the Diapason program. Qualitative results revealed little acceptance of the program and high expectations from caregivers. The Diapason program needs to evolve toward dynamic, flexible, and more customizable content based on a structure that favors interaction with professionals and peers, such as online community support [54].

### Conclusions

Although a lack of statistical power prevents any definitive conclusions being reached about the efficacy of this program, the mixed research analysis provided us with valuable information for improving content and methods. Caregivers outlined high expectations about the program’s functionalities and showed little acceptance of our program. Dynamism, flexibility, personalization, and socialization appeared as key characteristics expected by caregivers. Overall, further studies about caregivers’ help-seeking behaviors and readiness facilitators or predictors are warranted.

## Multimedia Appendix 1

CONSORT-EHEALTH checklist V1.6.2 [55].

## Abbreviations

BDI-II: Beck Depression Inventory-second version
BPSD: behavioral and psychological symptoms of dementia
IADL: Instrumental Activities of Daily Living
MMSE: Mini-Mental State Examination
NHP: Nottingham Health Profile
PSS-14: Perceived Stress Scale
PWAD: persons with Alzheimer’s disease
RCT: randomized controlled trial
RMBPC: Revised Memory and Behavior Problems Checklist
RSCS: Revised Scale for Caregiving Self-Efficacy
VAS: visual analog scales
ZBI: Zarit Burden Interview
