# Supplementary material for: A Web-Based Psychoeducational Program for Informal Caregivers of Patients With Alzheimer’s Disease: A Pilot Randomized Controlled Trial
Source: J Med Internet Res. 2015 May 12;17(5):e117. doi: 10.2196/jmir.3717 (PMC4468784; doi:10.2196/jmir.3717)
Supplement: Supplementary file 1 [file jmir_v17i5e117_app1.pdf]

CONSORT-EHEALTH Checklist V1.6.2 Report

(based on CONSORT-EHEALTH V1.6), available at [http://tinyurl.com/consort-ehealth-v1-6].

3717

Date completed

10/24/2014 11:06:11

by

Jérémy Wrobel

A Web-based psycho-educational program for informal caregivers of patients with Alzheimer's disease: a pilot randomized controlled trial.

TITLE

1a-i) Identify the mode of delivery in the title

Yes, our study evaluates a "web-based psycho-educational program"

1a-ii) Non-web-based components or important co-interventions in title

1a-iii) Primary condition or target group in the title

Yes, our program is destined for "informal caregivers of patients with Alzheimer's disease" and to evaluate the efficacity of the web-based program, we created two groups; the target group who could access to the program and the control group who could not.

ABSTRACT

1b-i) Key features/functionalities/components of the intervention and comparator in the METHODS section of the ABSTRACT

"Based on a face-to-face intervention program we adapted and designed a web-based fully automated psycho-educational program (called Diapason) inspired by a cognitive approach."
Experimental group has access to the Diapason program and "received the web-based intervention and the usual care during 3 months or only the usual care" for the control group. "Caregivers' perceived stress (PSS-14, primary outcome), self-efficacy, burden, perceived health status, and depression (secondary outcomes) were measured during three face-to-face onsite visits: at baseline, at the end of the program (M3), and after follow-up (M6). Additionally, semi-structured interviews were conducted with EG caregivers at M6, and treated with thematic analysis. "
"The Diapason program (www.etreaudiapason.com, was delivered in a free, password-protected, fully-automated website, to be used in an individual fashion, at home by the caregivers. The contents were focused on: a) caregivers' beliefs, about the illness and the caregiving role, b) caregivers' skills, to manage daily life difficulties, and c) caregivers' social support and help-seeking behavior to obtain respite or financial support, and to meet and discuss with peers through a forum. The program's content was grounded on cognitive theories of stress. Twelve thematic sessions were sequentially and weekly unblocked once the previous one was entirely visualized"

1b-ii) Level of human involvement in the METHODS section of the ABSTRACT

Yes, "The Diapason program was delivered in a free, password-protected, fully-automated website"

1b-iii) Open vs. closed, web-based (self-assessment) vs. face-to-face assessments in the METHODS section of the ABSTRACT

1b-iv) RESULTS section in abstract must contain use data

1b-v) CONCLUSIONS/DISCUSSION in abstract for negative trials

INTRODUCTION

2a-i) Problem and the type of system/solution

Yes, "due to the worldwide aging population, the number of persons with dementia (35.6 million currently) is expected to double by 2030." In addition to the cost, this represents an important hours for care usually provided by family number. "The physical efforts and the strong emotional involvement associated with caregiving may induce chronic stress in caregivers and weaken their physical and mental health"
"Various non-pharmacological intervention programs for caregivers are available onsite. Nevertheless, some caregivers are not willing or available to attend a face-to-face program due to a lack of respite, the distance or owing to care-recipients' behavioral or physical problems. For them, technology-based programs may represent an interesting complementary strategy to the regular care management"

2a-ii) Scientific background, rationale: What is known about the (type of) system

Yes, "due to a lack of respite, the distance or owing to care-recipients' behavioral or physical problems", caregivers cannot participate to a face-to-face program." For them, technology-based programs may represent an interesting complementary strategy to the regular care management"
" Although other recent Internet-based programs have been tested, to our knowledge the use of mixed research methods still remains rare in randomized controlled trials"

METHODS

3a) CONSORT: Description of trial design (such as parallel, factorial) including allocation ratio

Yes, "the main aim of this pilot RCT was to evaluate the impact of the Diapason program on caregivers' perceived stress. We hypothesized that this program, offering information, skills training and a forum for caregivers, would significantly reduce their perceived stress and burden, and enhance caregivers' self-efficacy, self-perceived health and self-perceived knowledge about the disease. Qualitative analyses would facilitate the identification of subgroups benefiting from the program, and would guide us to improve contents and methods to evaluate this type of interventions."

3b) CONSORT: Important changes to methods after trial commencement (such as eligibility criteria), with reasons

For this study, the item is not applicable because we did not conduct any "modification regarding methodology, program content (except for forum discussions) or website during the course of the study".

3b-i) Bug fixes, Downtimes, Content Changes

4a) CONSORT: Eligibility criteria for participants

Yes, "eligible participants were required to be French-speaking caregivers of community-dwelling AD patients who met the criteria of the Diagnostic and Statistical Manual of Mental Disorders, 4th Edition. Caregivers had to spend at least 4 hours per week with their relative, to be aged 18 or more, to score 12 or more on the Perceived Stress Scale (PSS-14), and to have access to a computer with Internet connection. Professional caregivers were ineligible."

4a-i) Computer / Internet literacy

4a-ii) Open vs. closed, web-based vs. face-to-face assessments:

"Recruitment strategy included flyers and posters placed in the hospital. During the consultations, geriatricians proposed this protocol to caregivers of PWAD. The caregivers interested by the study fulfilled a contact form.
Once validated the inclusion criteria and randomization performed, the participant received two face to face assessments. "Each 90-minute assessment visit consisted of a structured interview, standardized questionnaires and visual analogical scales. Additionally, EG volunteers participated in an optional one-to-one semi-structured interview at M6."
"Participants were recruited and randomized offline in two parallel groups, based on a computer-generated randomization list, using blocking and stratification by sex and relationship (spouses vs. non-spouses)." (...) " Once a week, participants had to read through one entire thematic session and fulfill a printed satisfaction questionnaire"
On the website, participants used nicknames to protect their privacy.

4a-iii) Information giving during recruitment

4b) CONSORT: Settings and locations where the data were collected

Yes, "at each visit (at the Broca Hospital) we collected information on caregiving variables (structured questionnaire)."
"Web metrics (session length and rate of visits) were collected for each EG participant automatically and anonymously. Participants completed a weekly satisfaction questionnaire focused on utility, clarity, and comprehensiveness (5-Likert scale). They rated from 0 to 100 the applicability and positive emotional impact of each session and reported their opinion of the program (open-ended question). At the end of their participation, we proposed a semi-structured interview exploring their opinion of the program.
Concerning the PWAD we collected at M0: Mini-Mental State Examination (MMSE) [31] from the medical record, and Instrumental Activities of Daily Living (IADL[32]) and the date of symptom onset (reported by the caregiver). "

4b-i) Report if outcomes were (self-)assessed through online questionnaires

Outcomes provide from standardized questionnaires filled out during the individual face-to-face assessment.
"Each 90-minute assessment visit consisted of a structured interview, standardized questionnaires and visual analogical scales. "
'Once a week, participants had to (...) fulfill a printed satisfaction questionnaire"

4b-ii) Report how institutional affiliations are displayed

5) CONSORT: Describe the interventions for each group with sufficient details to allow replication, including how and when they were actually administered

5-i) Mention names, credential, affiliations of the developers, sponsors, and owners

5-ii) Describe the history/development process

5-iii) Revisions and updating

5-iv) Quality assurance methods

5-v) Ensure replicability by publishing the source code, and/or providing screenshots/screen-capture video, and/or providing flowcharts of the algorithms used

5-vi) Digital preservation

5-vii) Access

"The Diapason program (www.etreaudiapason.com, was delivered in a free, password-protected, fully-automated website, to be used in an individual fashion, at home by the caregivers."
"Durint the recruitment, the experimental group’s participants received (at M0) a 10-minute training to use the website, a login and password" to connect from home.

5-viii) Mode of delivery, features/functionalities/components of the intervention and comparator, and the theoretical framework

Yes, the psycho-educational program is inspired by the group intervention sessions from the geriatric service of Broca Hospital to informal caregivers of persons with dementia. We
adapted the face-to-face intervention to an internet-delivered version.
"The contents were focused on: a) caregivers' beliefs, about the illness and the caregiving role, b) caregivers' skills, to manage daily life difficulties, and c) caregivers' social support and help-seeking behavior to obtain respite or financial support, and to meet and discuss with peers through a forum. The program's content was grounded on cognitive theories of stress. Twelve thematic sessions were sequentially and weekly unblocked once the previous one was entirely visualized."
"Each session included theoretical and practical information, videos of health professionals, and a practice guide for applying the session's content in real life. The length of the intervention was 3 months, with each weekly session lasting individually 15 to 30 minutes on average, but there was no time limit and the participants could access different website sections (e.g. relaxation training, forum and hyperlinks to other resources) for as long as they wished at any time. "
"Other website sections (eg. relaxation training, forum) were available but not mandatory to validate the program"

5-ix) Describe use parameters

5-x) Clarify the level of human involvement

5-xi) Report any prompts/reminders used

No automatic system for reminding sessions has been established in this study. However, participant received feedback on the main page wheter he validated/or not the session.

5-xii) Describe any co-interventions (incl. training/support)

Yes, "the CG and EG participants received usual care, in which they were provided with information on the illness during the geriatric semiannual follow-up. " "All participants were advised to look for additional help if necessary, and were asked to inform the researcher about it."

6a) CONSORT: Completely defined pre-specified primary and secondary outcome measures, including how and when they were assessed

Primary and secondary outocomes were assessed during the face-to-face assessment.
"To evaluate the perceived stress of caregivers (primary outcome), we used the 14-item Perceived Stress Scale (PSS-14). The secondary outcomes were (a) self-efficacy, measured by the Revised Scale for Caregiving Self-efficacy (RSCS)], (b) perception and reaction to cognitive or behavioral symptoms of PWAD were evaluated by the Revised Memory and Behavior Problems Checklist (RMBPC), (c) subjective burden was evaluated with the French version of the Zarit Burden Interview, (d) depressive symptoms were measured with the second version of the Beck Depression Inventory (BDI) including 21 items, and (e) self-perceived health was measured with the French version of the Nottingham Health Profile (NH]. We analyzed social isolation, emotional reactions, and sleep quality sub-scores, and rated each from 0 to 100, which provided a percentage of the perceived illness impact."

6a-i) Online questionnaires: describe if they were validated for online use and apply CHERRIES items to describe how the questionnaires were designed/deployed

6a-ii) Describe whether and how "use" (including intensity of use/dosage) was defined/measured/monitored

6a-iii) Describe whether, how, and when qualitative feedback from participants was obtained

6b) CONSORT: Any changes to trial outcomes after the trial commenced, with reasons

For this study, the item is not applicable because we have not made any changes to trial outcomes after the trial commenced: " any "modification regarding methodology, program content (except for forum discussions) or website during the course of the study".

7a) CONSORT: How sample size was determined

7a-i) Describe whether and how expected attrition was taken into account when calculating the sample size

7b) CONSORT: When applicable, explanation of any interim analyses and stopping guidelines

No,the item is not relevant for our study because we did not conduct any interim analyses.

8a) CONSORT: Method used to generate the random allocation sequence

"Participants were recruited and randomized offline in two parallel groups, based on a computer-generated randomization list, using blocking and stratification by sex and relationship (spouses vs. non-spouses). "
Of the 49 participants, 24 allocated to the control group and 25 to the experimental group.

8b) CONSORT: Type of randomisation; details of any restriction (such as blocking and block size)

Yes, randomisation was conducted in two parallel group after blocking and stratification by sex and relationship.

9) CONSORT: Mechanism used to implement the random allocation sequence (such as sequentially numbered containers), describing any steps taken to conceal the sequence until interventions were assigned

For the random allocation sequence, we used a" computer-generated randomization list, using blocking and stratification by sex and relationship"

10) CONSORT: Who generated the random allocation sequence, who enrolled participants, and who assigned participants to interventions

For the random allocation sequence, we used a" computer-generated randomization list"

11a) CONSORT: Blinding - If done, who was blinded after assignment to interventions (for example, participants, care providers, those assessing outcomes) and how

11a-i) Specify who was blinded, and who wasn't

The item is not applicable for our study because it was an unblinded study.

11a-ii) Discuss e.g., whether participants knew which intervention was the “intervention of interest” and which one was the “comparator”

11b) CONSORT: If relevant, description of the similarity of interventions

The item is not applicable for our study because we compare our experimental group to a group control.

12a) CONSORT: Statistical methods used to compare groups for primary and secondary outcomes

Yes, "descriptive statistics (means and percentages) were calculated for caregivers' and PWAD's characteristics. Moreover, t-tests (or Mann-Whitney tests) and Spearman or polyserial correlations were used to assess associations between variables. After checking normality and homoscedasticity of primary outcome (PSS), we conducted an analysis of covariance (ANCOVA), controlling for regression to mean phenomenon and effects of potential confounders at baseline on primary outcome. All analyses were conducted using R Software for Windows (version 3.0.0)."

12a-i) Imputation techniques to deal with attrition / missing values

" The missing data within each scale were treated according to the recommendations of the literature, when available. Otherwise, simple mean imputation was used. The last observation carried forward method was used for dropped out participants. "

12b) CONSORT: Methods for additional analyses, such as subgroup analyses and adjusted analyses

in this study, we have made an exact Fisher test to evaluate the correlation between the relationship and the opinion about the program.
Moreover, "we conducted an analysis of covariance (ANCOVA), controlling for regression to mean phenomenon and effects of potential confounders at baseline on primary outcome".

RESULTS

13a) CONSORT: For each group, the numbers of participants who were randomly assigned, received intended treatment, and were analysed for the primary outcome

Yes, we randomized 49 participants. Of the 25 participants allocated to experimental group, 17 (70.83%) finished the protocol and validated at least 10 of the 12 online sessions. Four participants ended their participation in the study without withdrawing consent."
Of the 24 participants allocated to control group, 17 finished the protocol and data analysed for the primary outcome.

13b) CONSORT: For each group, losses and exclusions after randomisation, together with reasons

Experimental Group: 25 participants
- 5 participants lost to M3: 2 stopped participations, 1 caregiver hospitalized and 2 became illegible
- 3 participants lost to follow up M6: 1 stopped participation, 1 patient hospitalized and 1 became illegible

Control group: 24 participants
- 4 participants lost to M3: 1 stopped participation, 2 patients institutionalized and 1 became illegible
- 3 participants lost to follow up M6: 2 patients hospitalized and 1 caregiver hospitalized

13b-i) Attrition diagram

14a) CONSORT: Dates defining the periods of recruitment and follow-up

The recruitment began in October 2011 and finished in July 2014.
When a participant were recruited, we organized a face-to-face assessment 3 months later and another one to the follow up 6 months later.

14a-i) Indicate if critical “secular events” fell into the study period

14b) CONSORT: Why the trial ended or was stopped (early)

"After eight-month recruitment extension, the main investigators (ASR and VCL) stopped recruitment (in total 20 months), since the rate of inclusions did not exceed two persons per month on average. "

15) CONSORT: A table showing baseline demographic and clinical characteristics for each group

	Experimental Group N=25	Control Group N=24
Caregivers' characteristics		
Caregiver age, yrs., mean (SD1)	64.2 (10.3)	59.0 (12.4)
Female caregiver, n (%)	16 (64.0)	16 (66.7)
Children of PWAD2,3, n (%)	16 (64.0)	13 (54.2)
High level of education, n (%)	19 (76.0)	18 (75.0)
Middle level of education, n (%)	6 (24.0)	3 (12.5)
Living with the PWAD, n (%)	12 (48.0)	10 (41.7)
Visiting the PWAD daily, n (%)	4 (16.0)	2 (8.3)
Visiting the PWAD at least once per week, n (%)	9 (36.0)	9 (37.5)
Psychological/ psychiatric treatment, n (%)	3 (12.0)	2 (8.3)
Psychotropic treatment, n (%)	6 (24.0)	7 (29.2)
Caregivers with at least another source of stress different to caregiving (work, relationship, family)	18 (72.0)	14 (56)
Caregivers with at least one professional help, n (%)	18 (72.0)	18 (75.0)
Weekly hours of professional help, mean (SD)	26.7 (28.7)	8.2 (9.7)
Suffering from a chronic pathology, n (%)	9 (36.0)	8 (33.3)

Data from the table 1

15-i) Report demographics associated with digital divide issues

Yes, to participate to this study, participants had to have access to a computer with internet connection.
"Only one user reported problems watching the videos (lacked installation of Flashplayer®) and another with little experience using the Internet could not use it unaided."

16a) CONSORT: For each group, number of participants (denominator) included in each analysis and whether the analysis was by original assigned groups

16-i) Report multiple "denominators" and provide definitions

We defined use of intervention de validation of at least 10 sessions from 12.

We did not have multiple denominators but only one to know the entire cohort because "All available data at baseline were analyzed by intention-to-treat"

16-ii) Primary analysis should be intent-to-treat

Yes, " All available data at baseline were analyzed by intention-to-treat"

17a) CONSORT: For each primary and secondary outcome, results for each group, and the estimated effect size and its precision (such as 95% confidence interval)

For the primary outcome (Self Perceived Stress), T-tests (or Mann-Whitney tests) did not show significant differences between experimental group and control group over time. We conducted ANCOVA with PSS-14 at M3 as dependent variable, and PSS-14 at M0, group, stratification factors (sex and relationship), and potential confounders at M0 (BDI and professional help received) as independent variables. Only PSS-14 at baseline (P<0.001) and weekly help received (P=.013) were significantly associated with PSS-14 at M3. Thus, no significant relationship was found with the intervention (P=0.3). ANCOVA showed similar results when stratification factors were not included.

17a-i) Presentation of process outcomes such as metrics of use and intensity of use

17b) CONSORT: For binary outcomes, presentation of both absolute and relative effect sizes is recommended

This item is not relevant for our study because our "outcome" is the perceived stress measured on a continuous scale. We did not have binary outcomes.
For the absolute effect sizes, we used to Cohen's d.

18) CONSORT: Results of any other analyses performed, including subgroup analyses and adjusted analyses, distinguishing pre-specified from exploratory

For this study, we did not conduct subgroup analyses because our sample was so small.
However, " we conducted ANCOVA with PSS-14 at M3 as dependent variable, and PSS-14 at M0, group, stratification factors (sex and relationship), and potential confounders at M0 (BDI and professional help received) as independent variables. Only PSS-14 at baseline (P<0.001) and weekly help received (P=.013) were significantly associated with PSS-14 at M3. Thus, no significant relationship was found with the intervention (P=0.3). ANCOVA showed similar results when stratification factors were not included.

18-i) Subgroup analysis of comparing only users

19) CONSORT: All important harms or unintended effects in each group

Yes "Only one user reported problems watching the videos (lacked installation of Flashplayer®) and another with little experience using the Internet could not use it unaided."

19-i) Include privacy breaches, technical problems

19-ii) Include qualitative feedback from participants or observations from staff/researchers

DISCUSSION

20) CONSORT: Trial limitations, addressing sources of potential bias, imprecision, multiplicity of analyses

20-i) Typical limitations in ehealth trials

Yes, "In spite of different strategies, the recruitment of this study was difficult, only 37.98% (49/129) of pre-screened caregivers were actually enrolled. These difficulties are evoked in Internet-based intervention studies, suggesting it may be due to caregivers' attitudes towards these programs"
We could not modify the content during the study and particpants would have like to have flexible content with updates. "The Diapason program needs to evolve towards a dynamic, flexible, and more customizable content, based on a structure which favors interaction with professionals and peers, such as online community support"

21) CONSORT: Generalisability (external validity, applicability) of the trial findings

21-i) Generalizability to other populations

21-ii) Discuss if there were elements in the RCT that would be different in a routine application setting

22) CONSORT: Interpretation consistent with results, balancing benefits and harms, and considering other relevant evidence

22-i) Restate study questions and summarize the answers suggested by the data, starting with primary outcomes and process outcomes (use)

Due to the low statistical power, any significant differences between the groups were showed with the statistical analysis. "Perceived stress levels remained stable over time in PSS although AD progressed "

22-ii) Highlight unanswered new questions, suggest future research

Other information

23) CONSORT: Registration number and name of trial registry

Trial Registration: Clinicaltrials.gov NCT01430286; http://clinicaltrials.gov/ct2/show/NCT01430286 (WebCite at http://www.webcitation/6KxHaRspL).

24) CONSORT: Where the full trial protocol can be accessed, if available

"the in-depth description of protocol study has been reported in this article http://www.researchprotocols.org/2013/2/e55/
And http://clinicaltrials.gov/ct2/show/NCT01430286 (WebCite at http://www.webcitation/6KxHaRspL).

25) CONSORT: Sources of funding and other support (such as supply of drugs), role of funders

"This study was partially funded by the French Health Ministry (Projet de Recherche en Qualité Hospitalière 2009–PREQHOS 2009) and by the Fondation Méderic Alzheimer project grants 2012-2014. "

X26-i) Comment on ethics committee approval

x26-ii) Outline informed consent procedures

X26-iii) Safety and security procedures

X27-i) State the relation of the study team towards the system being evaluated
